# Molecular Aspects of the Emergence of Monkeypox Virus Clades

**DOI:** 10.3390/v17121549

**Published:** 2025-11-26

**Authors:** Igor V. Babkin, Irina N. Babkina, Nina V. Tikunova

**Affiliations:** 1 Laboratory of Molecular Microbiology, Institute of Chemical Biology and Fundamental Medicine Siberian Branch of the Russian Academy of Sciences, Novosibirsk 630090, Russia; 2 AO Vector-Best, Novosibirsk 630117, Russia; irinanik_babkina@mail.ru

**Keywords:** monkeypox virus, mpox, orthopoxvirus, adaptive selection, evolution, mutation accumulation rate, origin

## Abstract

Monkeypox virus (MPXV), which previously caused mainly zoonotic infections, is currently the causative agent of the mpox outbreak that began in 2022. Since the mpox outbreak is characterized by sustained human-to-human transmission, the evolutionary trajectory of MPXV is an important scientific issue. The prevailing hypothesis suggests that the modern orthopoxviruses originated from cowpox-like ancestors with larger genomes that infected a wide range of hosts. Subsequent evolution included the reduction of the genome and the accumulation of substitutions in key proteins. Molecular dating of MPXV evolution revealed 5–6-fold acceleration in the evolutionary rate that was observed in subclade IIb after 2018, reaching 1.8 × 10^−5^ substitutions/site/year, likely due to virus’ adaptation to humans. The origin of MPXV from its precursor was primarily driven by the accumulation of non-synonymous substitutions in the key host range genes, including those associated with the protein inhibiting host protein synthesis (OPG173) and host immune evasion (OPG027). The subsequent divergence of MPXV into clades I and II largely depended on mutations in the gene encoding the Bcl-2-like protein. Finally, the division of clade II into subclades IIa and IIb was facilitated by further non-synonymous substitutions in the soluble interferon alpha/beta receptor and hemagglutinin genes.

## 1. Introduction

Monkeypox virus (MPXV), a member of the Orthopoxvirus genus, is a causative agent of mpox, a disease with symptoms similar to smallpox but usually not so severe [1,2,3]. The virus was first identified in 1958 in monkeys kept in a Danish laboratory, hence the name “monkeypox” [4,5,6]. The first human case was reported in an infant in the Democratic Republic of the Congo (DRC) in 1970 [4,7,8]. It has been discussed for a long time whether MPXV is a separate species or a subtype of the variola virus (VARV). The relevance of this discussion was supported by the possible existence of natural smallpox reservoirs; however, studies have shown significant genetic differences between MPXV and VARV. Sequencing of the orthopoxvirus (VARV and MPXV) genomes confirmed the assignment of MPXV to a separate virus species [9,10,11,12].

MPXV has a linear double-stranded DNA genome of approximately 200 kb with a central highly conserved core region ~101 kb containing genes that are vital for the virus [1,5]. Most of the key proteins encoded by the genes from the central conservative region are involved in the main stages of virus morphogenesis: DNA replication and repair, transcription, protein modification, and virion formation. The central region of the MPXV genome is flanked by the extended terminal variable regions with inverted terminal repeats at their ends, which predominantly harbor genes associated with the host range and immunomodulation [1,5,13,14].

Based on the genomic sequences of MPXV isolates, two genetic clades have been distinguished: clade I (former Central African clade) and clade II (former West African clade) [15,16]. These clades have been further subdivided into subclades Ia, Ib and IIa, IIb [17,18]. Before the 2022 outbreak, mpox caused by clade I MPXV was associated with a case fatality rate (CFR) exceeding 10%, whereas infection caused by clade II MPXV was usually mild, with a CFR of less than 4% within Africa [19,20,21].

For decades, sporadic, localized outbreaks of monkeypox resulting from zoonotic transmission from wildlife to humans, have been reported in Central and West Africa [22,23,24]. In a number of cases, this infection has been carried to other continents; however, imported infections have not been widespread in these years [25,26,27,28]. From May 2022 to the present, there has been an unprecedented large-scale epidemic of mpox. A total of 124,753 laboratory confirmed cases in 128 countries have been reported to the World Health Organization (WHO) from 2022 to 2024, including 272 deaths (available online: https://worldhealthorg.shinyapps.io/mpx_global/#1_Overview; accessed on 11 June 2025). The outbreak, driven by subclade IIb MPXV, exhibited a CFR significantly below 0.1% [2] (available online: https://worldhealthorg.shinyapps.io/mpx_global/#section-fns2; accessed on 1 July 2025). In 2024, a distinct subclade, Ib, was identified in the DRC. Similarly to subclade IIb, subclade Ib MPXV demonstrated efficient human-to-human transmission and have spread to neighboring countries, causing a new epidemic wave. Imported cases of subclade Ib MPXV have been reported in the United States, Europe (Germany, Sweden, and the United Kingdom), and Asia (China, India, and Thailand). The CFR associated with subclade Ib infection remains an open question; to date, no deaths have been reported outside Africa [29] (available online: https://www.who.int/ru/news/item/28-11-2024-second-meeting-of-the-international-health-regulations-(2005)-emergency-committee-regarding-the-upsurge-of-mpox-2024; accessed on 11 June 2025). This evolving epidemiology underscores questions about the virus’s ongoing evolutionary trajectory.

Unlike VARV, which is a strictly anthroponotic virus, MPXV infects a wide range of sensitive hosts. Despite the fact that the exact host of MPXV is still unknown, it seems that it is mainly spread by rodents in the wild [6,30,31]. Before 1980, 91% of infections were attributed to contact with infected animals [32]; outbreaks after 1980 showed an increasing proportion of human-to-human transmission [33,34], though zoonotic spillover events remained the primary trigger [35]. The mpox outbreak that began in 2022 was characterized by sustained human transmission, mainly through close contacts [2]. To assess the potential of transmission, the basic reproduction number R_0_, is used, a key epidemiological metric, where R_0_ exceeding 1 indicates the potential for epidemic spread. Estimates of R_0_ for clade I MPXV in the DRC during 1980–1984 were approximately 0.32 [35]. In contrast, R_0_ for the 2022 clade IIb outbreak was estimated at 2.44 in Europe, though this high transmission rate was mainly limited to men who had sex with men and not the general population [36]. A significant additional risk associated with the global mpox outbreak is the potential establishment of new animal reservoirs outside Africa. A case of human-to-dog transmission has been reported [37]. If the virus spills over and becomes endemic among local rodent populations in Eurasia and America, it could establish a permanent zoonotic reservoir on these continents, complicating long-term efforts to control this infection.

The global mpox epidemic highlights the critical need for epidemiological and genetic surveillance of MPXV isolates from both wildlife and humans. This study provides a comprehensive evolutionary analysis of available MPXV isolates including data on the ongoing epidemic. In addition, we conducted a comparative analysis of MPXV with the Kostroma strain of the cowpox virus (CPXV), which shares the most recent common ancestor with MPXV. This approach enabled the identification of the genes that underwent adaptive selection during the divergence of MPXV from its common ancestor with CPXV.

## 2. Materials and Methods

### 2.1. Retrieval of Genome Sequences and Alignment

Nucleotide sequences of orthopoxviruses were obtained from the NCBI database (available online: http://www.ncbi.nlm.nih.gov/; accessed on 1 June 2025). The highly conserved central genomic region flanked by the open reading frames (ORFs) F4L and A24R (according to the nomenclature of the Vaccinia virus Copenhagen strain) was extracted from the genome sequences of MPXV strains and aligned using MAFFT v.7 [38].

### 2.2. Phylogenetic Tree Construction and Evolutionary Analyses

An initial maximum likelihood (ML) tree was constructed using PhyML v.3.0 [39] to evaluate the presence of a temporal signal in the dataset. This was done by regressing root-to-tip genetic distances against sampling years using TempEst v.1.5.3 [40].

The phylogeographic analysis and molecular dating were performed using the Bayesian Markov chain Monte Carlo (MCMC) inference method implemented in BEAST 2 v.2.7.4 (Bayesian Evolutionary Analysis by Sampling Trees) software [41]. The analysis was run using a log-normal relaxed molecular clock model and a coalescent Bayesian skyline population prior. MCMC chains were run for 500 million steps to ensure convergence, with the first 10% discarded as burn-in. Proper mixing of the Markov chain was assessed by calculating the effective sample size (ESS) for all parameters using Tracer v1.7.2 software (part of the BEAST package). A maximum clade credibility tree was generated using the HKY substitution model with unequal base frequencies, a proportion of invariant sites, and gamma-distributed rate heterogeneity among sites. This model is known to reliably describe variation in coding genomic sequences [42].

Phylogenetic trees based on maximum likelihood were constructed using IQ-tree v.2.4.0 software [43], employing the optimal substitution model [44]. Branch support was evaluated with 500 bootstrap iterations.

### 2.3. Detection of Evolutionary Pressure

Tests for negative (purifying) and adaptive (positive, diversifying) selection were performed using the codon-based Z-test of adaptive selection based on the Nei–Gojobori method [45] within the MEGA 7 package [46], with the neutral model as the null hypothesis. This test distinguishes between adaptive and negative selection by performing pairwise comparisons of the relative frequencies of synonymous (dS) and non-synonymous (dN) substitutions. The null hypothesis (dN = dS) was tested against the alternative hypotheses of dN > dS (indicating adaptive selection) and dN < dS (indicating negative selection) using the one-tailed Z-test at a 95% significance level.

## 3. Results

### 3.1. Selection of Genome Sequences

**Figure 1:**
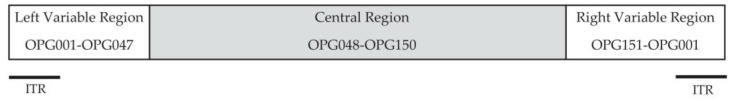
MPXV genome. ITR-inverted terminal repeats that are located in variable regions and contain ORFs, hairpin loops, and short tandem repeats.

This study was based on the available data on MPXV sequences. A total of 973 MPXV sequences were presented in the GeneBank database (September 2025) with a genome length of more than 190 kb. MPXV sequences with a fully sequenced central genome region were selected from this set of sequences. Then, the genomes with identical sequences in the central region were excluded and MPXV isolates with identical sequences were represented in the study by a single isolate. Sequences of the highly conserved central genome region flanked by the OPG048 and OPG151 (according to the Senkevich et al. nomenclature [47]) were determined (Figure 1) and used for evolutionary analysis, since it is known that recombination rearrangements that can interfere with phylogenetic studies [48] occur only in variable terminal regions of the orthopoxvirus genomes [49,50] with the exception of CPXV [51]. As a result, 206 MPXV sequences of the central conservative region, about 101 kb in length, containing the genes vital for orthopoxviruses, were selected for evolutionary analysis.

To determine whether the dataset was suitable for evolutionary analysis, the temporal structure of the data was evaluated. The initial maximum likelihood (ML) tree was constructed based on the multiple alignment of the MPXV genome central region that allowed to establish relationships between MPXV isolates (Figure S1). Then, TempEst v.1.5.3 was used to determine the time signal for the dataset and to identify outliers. ML phylogenetic analysis revealed two MPXV isolates with a significant loss of temporal signal: NC_003310_Zaire-96-I-16 and HM172544_Zaire_1979-005. It should be noted that the NC_003310_Zaire-96-I-16 strain was subcloned and sequenced by the Maxam-Gilbert method [11] that could result to some nucleotide substitution. The cidofovir-resistant strain HM172544_Zaire_1979-005 has a long passage history [52] that could have led to a large number of substitutions in their genomic sequences. Additional analysis of the ML phylogeny in the TempEst program without these two strains showed a correlation coefficient (slope rate) of 9.0 × 10^−6^ substitutions per site per year for the remaining 204 MPXV sequences. Thus, the dataset of 204 sequences was deemed suitable for molecular dating.

### 3.2. Molecular Dating in the Evolution of MPXV

**Figure 2:**
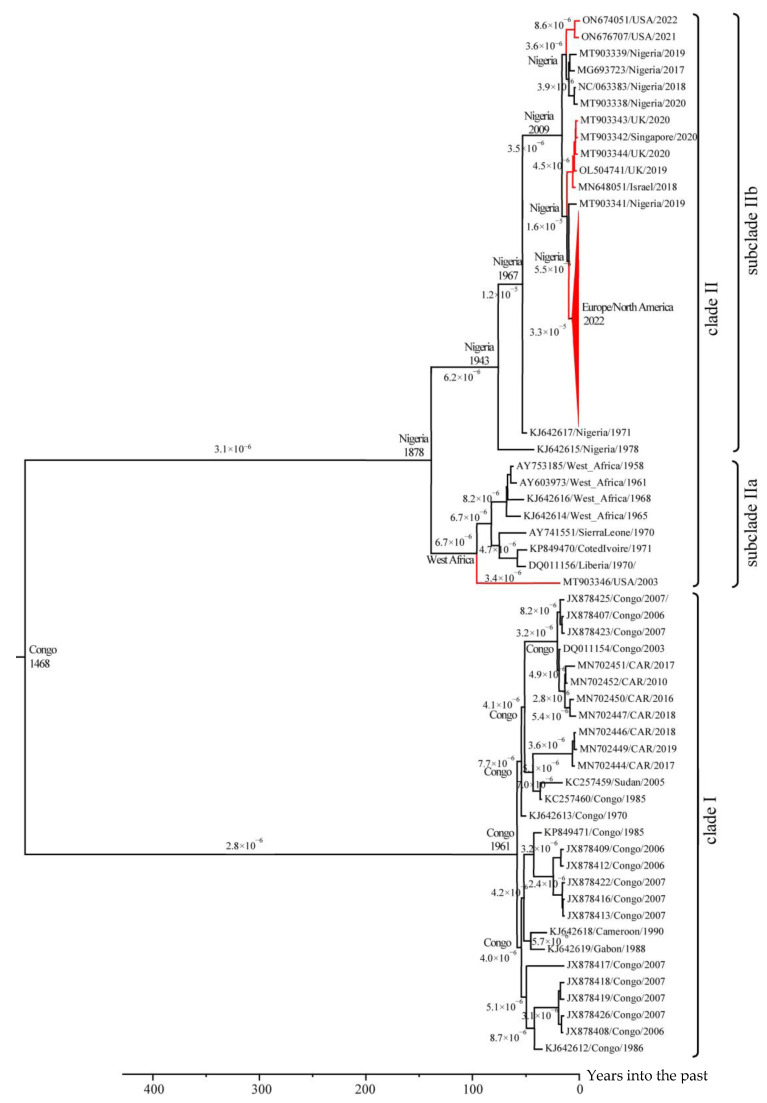
The phylogeographic analysis of MPXV. Maximum clade credibility tree for the highly conserved central genomic region of MPXV. The chronogram was generated using BEAST 2 v.2.7.4 software. A log-normal relaxed clock and coalescent Bayesian skyline population prior were used, as well as an HKY substitution model with unequal base frequencies, invariant sites, and gamma-distributed rate heterogeneity among sites. Taxon names indicate: GenBank accession number, region of sequence origin, and collection date. The rates of mutation accumulation are shown near the branches (substitutions/site/year). The tree nodes show the date and area of origin of the clade. Events of MPXV exported from Africa to other continents are highlighted in red.

Based on the 204 selected unique sequences of the central conserved region of the MPXV genome, an evolutionary phylogeographic analysis was performed using lognormal relaxed clocks with a coalescent Bayesian skyline population model in the BEAST 2 v.2.7.4 program. The use of this time estimates and the collection dates of various MPXV isolates allowed us to conduct an evolutionary study of an extended set of MPXV isolates (Figure 2). The study confirmed that the West African genotype (clade II) separated from the common ancestor with clade I in Central Africa in the 15th century [53]. Molecular dating of MPXV evolution revealed that the average rate of mutation accumulation in the MPXV genome was 3.3 × 10^−6^ substitutions per site per year. In the 19th century in Nigeria, clade II MPXV diverged into two subclades showing significant genetic differences: one subclade includes strains from Nigeria and strains from other continents (subclade IIb), and the other subclade contains strains from the rest of West Africa (subclade IIa). Phylogenetic analysis revealed that at least three cases of export of clade IIb MPXV isolates from Nigeria to other continents occurred between 2018 and 2022. One export event of clade IIa MPXV isolate occurred in 2003 from West Africa (Ghana), as it was confirmed by epidemiological data [24,54,55,56].

### 3.3. Genome Content and Adaptive Selection

**Figure 3:**
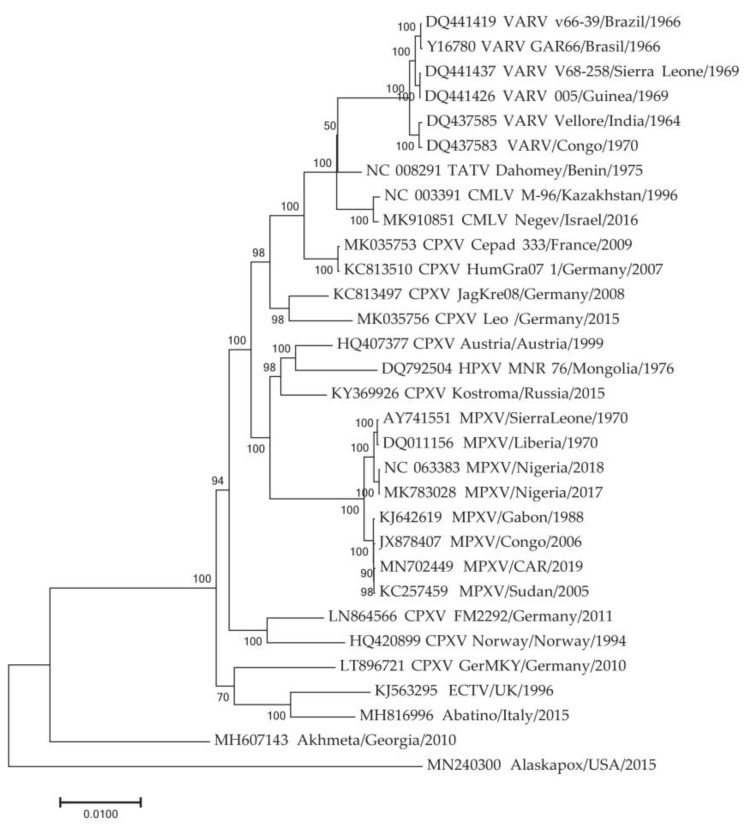
Phylogenetic tree for the highly conserved central genome regions of the orthopoxviruses was generated using the maximum-likelihood method. TATV—Taterapox virus, CMLV—Camelpox virus, HPXV—Horsepox virus, ECTV—Ectromelia virus. Numbers above and under branches indicate bootstrap support (%). Divergence (substitutions per site) scales are given at the bottom.

The phylogeographic analysis was based on the central conserved region of the MPXV genome. However, when MPXV adapts to new hosts, the main evolutionary changes occur in the extended variable terminal regions of the viral genome. It has been previously shown that some genes in these regions are under adaptive selection [13,57]. To identify these genes, the genome of the closest relative to MPXV was determined. A phylogenetic analysis of the sequences of Old World orthopoxviruses indicated that the genome of the CPXV strain Kostroma_2015 [58] is the closest to the ancestral MPXV sequence (Figure 3). It has been previously shown that MPXV diverged from a common ancestor with CPXV Kostroma_2015 approximately 3.5 thousand years ago [53]. Next, the complete genomes of 57 MPXV sequences representing the various clusters of the phylogram (Figure 2) and the genome of the CPXV Kostroma_2015, were aligned.

A pairwise comparison of 112 genes from the MPXV terminal variable regions with those of CPXV strain Kostroma_2015 (Tables S1 and S2) revealed the existing gene repertoires of each MPXV isolate. It should be noted that during the speciation of MPXV from a common ancestor with CPXV, partial reduction of the MPXV genome and inactivation of some MPXV genes occurred. Thirty-five ORFs were disrupted in all analyzed MPXV genomes studied (Table 1). These ORFs encoded seven ankyrin repeat-containing proteins, six kelch-like proteins, six TNF-receptor-like proteins, three IL-binding proteins, three Bcl-2-like proteins, three C-type lectin family proteins, the apoptosis regulator protein, guanylate kinase, immunoprevalent protein, semaphorin-like protein, and three other proteins. Within the MPXV species, differences in the repertoire of genes from the variable genomic regions between different virus genotypes are evident. For example, in clade I OPG003 and OPG016 (Senkevich et al. nomenclature [47]) are disrupted, encoding an ankyrin repeat-containing protein and an MHC class I-like protein, respectively. It should be noted that the OPG016 is also absent in subclade IIb. In MPXV clade II, OPG032, encoding a secreted complement-binding protein, is disrupted. In subclade IIa, OPG195 and OPG201, encoding an ER-localized apoptosis regulator and an IL-1-beta-binding protein, respectively, are additionally absent. Moreover, the UTC isolate of MPXV from subclade IIa lacks OPG039 (ankyrin-like protein) (Table 1).

**Figure 4:**
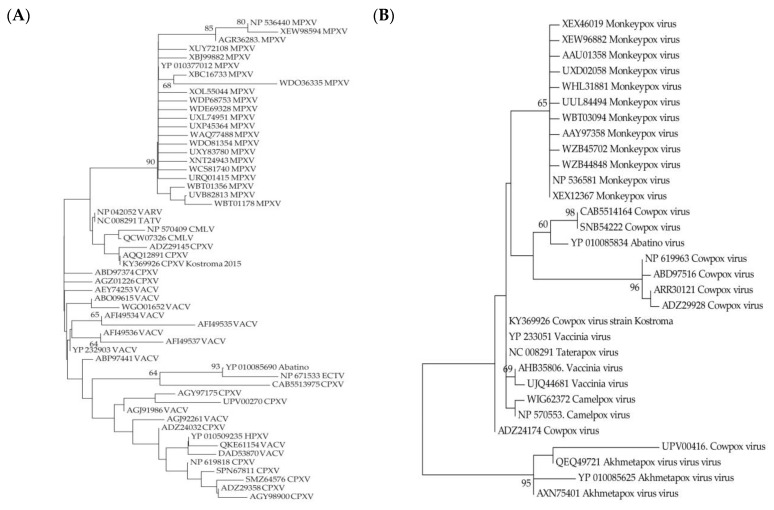
Maximum likelihood phylogenetic trees of the Old World orthopoxviruses proteins encoded by OPG027 (**A**) and OPG173 (**B**). TATV—Taterapox virus, CMLV—Camelpox virus, HPXV—Horsepox virus, ECTV—Ectromelia virus, VACV—vaccinia virus. Numbers above and under branches are bootstrap support (%).

One way to determine the type of evolutionary selection influencing on a gene is to compare the relative numbers of synonymous and non-synonymous substitutions that have occurred in the gene sequence. Tests for negative and adaptive selection were performed using the neutral model as a null hypothesis for the genes from the variable terminal regions preserved in the genomes of MPXV and CPXV Kostroma_2015 (Table 2). For most of the CPXV_Kostroma_2015 genes, negative selection was shown relative to MPXV isolates genes. At the same time, adaptive selection was revealed for the host-range protein gene (OPG027) and the gene of protein inhibiting host protein synthesis (OPG173) (Table 2). Host-range proteins encoded by OPG027 are conservative in orthopoxviruses and bind human antiviral factor SAMD9, which allows overcoming barrier posed by the host innate immune system [59]. As for proteins encoded by OPG173, they are conservative in CPXV, MPXV, Camelpox, Taterapox, Abatino, Ahmetapox viruses and some vaccinia virus (VACV) strains and are absent in other orthopoxviruses. These proteins inhibit translation initiation, which affects the outcome of orthopoxvirus infection by reducing the number of inflammatory leukocytes and decreasing the response of CD8+ memory T cells [60]. A comparative analysis of the MPXV proteins encoded by the OPG027 and OPG173 genes with other Old World orthopoxvirus proteins from nr GenBank database showed that MPXV proteins form separate clades on both trees and differ significantly from other orthopoxviral proteins (Figure 4).

When comparing all isolates of MPXV clades I and II, an adaptive selection was revealed for OPG044, whereas an adaptive selection was revealed for OPG163, OPG191, OPG198, OPG209, OPG210, and OPG003 when comparing some MPXV isolates. Within clade I, adaptive selection for OPG047 was shown between some isolates. In the case of OPG031 and OPG164, adaptive selection was observed for clade I isolates relative to some clade II isolates (Table 2).

When comparing subclades IIa and IIb MPXV for all isolates, a statistically significant adaptive selection was revealed for OPG185 (hemagglutinin) and OPG204 (soluble interferon alpha/beta receptor). In the case of OPG161 and OPG208, adaptive selection was defined for some isolates of clade IIa compared to clade IIb. Adaptive selection for OPG023, OPG025, OPG045, OPG152, OPG153, and OPG172 was also revealed for individual isolates within clade IIa (Table 2).

## 4. Discussion

The ongoing mpox outbreak underscores the importance of studying orthopoxvirus evolution. The molecular evolution of modern MPXV indicates a significantly increased mutation accumulation rate, leading to more efficient person-to-person transmission [76]. The current outbreak is caused by the West African genotype (clade II) of MPXV, which is associated with low mortality. However, there is evidence that the more virulent Central African virus (clade I) has evolved towards greater human transmissibility [77]. The adaptation to human-to-human transmission, coupled with the wide range of susceptible animal species and the emergence of low-virulence MPXV strains, facilitated its global spread. An additional contributing factor was the social stigma associated with the disease.

The average rate of substitution accumulation in the MPXV genome was 3.3 × 10^−6^ substitutions per site per year. However, after 2018, the evolutionary rate of subclade IIb (Nigerian lineage) in Europe and America, calculated based on the central conserved region of the genome, increased 5–6 fold, reaching 1.8 × 10^−5^ substitutions per site per year (Figure 2). This increase is likely related to viral adaptation to a new host. The more virulent clade I MPXV strains continue to evolve, with modern isolates forming several distinct groups. Their further evolution may lead to the emergence of new MPXV genotypes. Currently, most clade I MPXV isolates evolve at a rate of approximately 3–4 × 10^−6^ substitutions per site per year, although this rate increases to 8 × 10^−6^ for some isolates (Figure 2). However, as demonstrated by subclade IIb, a large-scale outbreak in the human population can significantly increase the evolutionary rate.

As previously mentioned, MPXV is pathogenic to a wide range of mammals, with rodents presumed to be the primary reservoir hosts [30,31]. The names “monkeypox virus” and “cowpox virus” are misnomers, as both are effective rodent poxviruses. Humans, monkeys, and cows are incidental hosts, and until recently, these hosts were unable to sustain MPXV circulation in the wild. The large-scale mpox epidemic has driven the adaptation of subclade IIb MPXV to humans, facilitating rapid evolutionary changes in the viral genome. It is plausible that the emergence of MPXV clades I and II, and their subsequent divergence into subclades IIa and IIb, is linked to past adaptations to new hosts. We conclude that different genes have contributed variably to both MPXV speciation and the formation of its genotypes at different phylogenetic levels. A relatively small number of genes appear to undergo adaptive selection. The primary mechanism of adaptation to host immune selective pressure involves changes in the gene content within the terminal variable regions of the MPXV genome. The process of evolutionary change in the MPXV genome is currently accelerating, leading to the emergence of numerous genetic variants with mutations disrupting various ORFs [78].

The divergence of MPXV into clades I and II likely had geographical drivers: clade I is prevalent in the Congo River basin, while clade II is found in the Niger River basin. These two regions are separated by a mountain range. Differences in the gene repertoire of the terminal variable regions between these clades are notable and can contribute to their differing CFR. For instance, genes encoding MHC class I-like protein and ankyrin repeat-containing proteins and are disrupted in clade I MPXV, whereas the gene encoding the secreted complement-binding protein is disrupted in clade II.

Our results in identifying genes under adaptive selection are consistent with the previously obtained data. McLysaght et al. [57], using the complete genomes of 20 poxviruses of various genera, showed that a number of genes are under adaptive selection; in particular, they noted that OPG185 (hemagglutinin,) is under adaptive selection. Esteban and Hutchinson [13] studied genes located in the terminal regions of orthopoxvirus genomes that are under adaptive molecular evolution. They showed with high confidence that 25 genes undergo adaptive selection. This type of selection was shown for OPG185 (hemagglutinin), OPG044 (Bcl-2-like protein), OPG045 (caspase-9 inhibitor), and OPG198 (Ser/Thr kinase protein). Molteni et al. [79] searched for adaptive selection when comparing the reference genomes of various orthopoxviruses and identified genes targeted by adaptive selection. In particular, they showed that OPG185 (hemagglutinin) and OPG208 (serine protease inhibitor) are under adaptive selection.

It can be concluded that the accumulation of non-synonymous substitutions in the genes for the host-range protein, which is likely involved in host immune evasion (OPG027, C7L according to the nomenclature of the Vaccinia virus Copenhagen strain) and the protein inhibiting host protein synthesis (OPG173), played an important role in the origin of MPXV from its precursor virus. The orthopoxvirus genomes contain a unique set of genes encoding proteins that block the host’s antiviral defense system and are responsible for immune evasion strategies. The protein encoded by OPG027, whose target is SAMD9 [59], is present in all orthopoxviruses and an interesting fact is that the sequence of this MPXV protein is the closest to VARV proteins (Figure 4). The protein encoded by OPG173 is absent in VARV and its function of inhibiting innate immune response [60] is probably taken over by other proteins.

In the subsequent evolution of MPXV, leading to the division of clades I and II, mutations in the gene encoding a Bcl-2-like protein (OPG044, K7R according to the nomenclature of the Vaccinia virus Copenhagen strain) was essential. This protein promotes virulence by binding to the host TRAF6 (TNF Receptor-Associated Factor 6) and IRAK2 (Interleukin-1 Receptor-Associated Kinase 2) and preventing host NF-kappa-B activation. Then, non-synonymous substitutions in the hemagglutinin (OPG185) and soluble interferon alpha/beta receptor (OPG204) genes played an important role in the separation of MPXV clade II into subclades IIa and IIb. It should be taken into account that results obtained when comparing viral sequences belonging to the same population (within a virus subclade with a common geographical distribution area) may be less reliable, as differences between sequences may represent segregating polymorphisms [80]. All of the above proteins are not included in either extracellular enveloped virus or intracellular mature virus, with the exception of the viral hemagglutinin [81,82], which is a glycoprotein found on the envelope of extracellular virus [83,84].

Orthopoxviruses, including MPXV, possess large double-stranded DNA genomes and accumulate mutations slowly. The evolutionary history of Old World orthopoxviruses (with the exception of Alaskapox and Akhmeta viruses) suggests they originated from a CPXV-like ancestor. CPXV has the largest and most diverse genome among orthopoxviruses, ranging from 220 to 230 kb. This diversity is so extensive that it may be more accurate to classify them as separate species rather than under the unified name “cowpox virus” [51,85]. CPXV typically causes mild infection in susceptible mammals. Other orthopoxvirus species, including MPXV, appear to have evolved from CPXV-like precursors through mutational changes and, primarily, genome reduction, losing immunomodulatory genes located in the terminal variable regions. This process gave rise to highly specialized and often highly virulent viruses such as VARV (smallpox), camelpox, ectromelia, and taterapox viruses, as well as MPXV, vaccinia, and Abatino viruses [53]. The genomes of VARV (~185 kb) and MPXV (~200 kb) are 30–40 kb smaller than the CPXV genome [1]. The emergence of new orthopoxvirus species typically requires hundreds of years. However, viral evolution can accelerate during large-scale epidemics, as witnessed during the 2022 global mpox outbreak.

All known CPXV isolates were obtained in Europe. However, evolutionary analysis indicates that MPXV originated from a CPXV-like ancestor on the African continent, while VARV emerged in the Middle East [53]. It is possible that CPXV genotypes genetically similar to MPXV and other orthopoxviruses exist or existed in these regions; however, this question requires further investigation.

Studying viral genome variability is crucial for assessing the epidemic potential of emerging viral variants and for predicting the emergence of new infectious agents, which will inevitably arise as viruses continue to evolve.

## Figures and Tables

**Table 1:** List of cowpox virus strain Kostroma_2015 genes absent in MPXV.

#	Gene Name ^a^	Gene Name ^b^	Function	The Gene Is Missing in MPXV
1.	D3L	OPG003	ankyrin repeat-containing protein	clade I
2.	D4L	OPG004	ankyrin repeat-containing protein	all strains
3.	D5L	OPG005	Bcl-2-like protein	all strains
4.	D6L	OPG006	alpha-amanitin target protein	all strains
5.	D7L	OPG008	kelch-like protein	all strains
6.	D8L	OPG009	ankyrin repeat-containing protein	all strains
7.	D9L	-	C-type lectin-like protein	all strains
8.	D10L	-	C-type lectin domain-containing protein	all strains
9.	D11L	OPG011	kelch-like protein	all strains
10.	D12L	OPG012	TNF-alpha-receptor-like protein	all strains
11.	D13L	OPG013	TNF-alpha-receptor-like protein	all strains
12.	D14L	OPG014	ankyrin repeat-containing protein	all strains
13.	C2L	OPG016	MHC class I-like protein	clade I and IIb
14.	C3L	OPG017	ankyrin repeat-containing protein	all strains
15.	C4L	OPG018	host-range protein	all strains
16.	C6L	OPG020	IL-1 receptor antagonist	all strains
17.	C12L	OPG026	TNF-rcpt-II_C domain containing protein	all strains
18.	C17L	OPG032	secreted complement-binding protein	clade II
19.	C18L	OPG033	kelch-like protein	all strains
20.	C19L	OPG034	Bcl-2-like protein	all strains
21.	P1L	OPG037	ankyrin-like protein	all strains
22.	M1L	OPG039	ankyrin-like protein	strain UTC
23.	A41R	OPG168	semaphorin-like protein	all strains
24.	A42R	OPG169	C-type lectin-like type-II membrane protein	all strains
25.	A50L	OPG177	immunoprevalent protein	all strains
26.	A52R	OPG179	Bcl-2-like protein	all strains
27.	A55R	OPG182	Toll-IL receptor-like protein	all strains
28.	A56R	OPG183	secreted TNF-receptor-like protein	all strains
29.	A57R	OPG184	kelch-like protein	all strains
30.	A59R	OPG186	guanylate kinase	all strains
31.	B8R	OPG195	ER-localized apoptosis regulator	clade IIa
32.	B9R	OPG196	Kelch-like protein	all strains
33.	B14R	OPG201	IL-beta-binding protein	clade IIa
34.	B15L	OPG202	IL-1-beta-inhibitor	all strains
35.	B16R	OPG203	ankyrin-like protein	all strains
36.	B19R	OPG206	Kelch-like protein	all strains
37.	K1R	OPG211	ankyrin repeat containing protein	all strains
38.	K2R	OPG212	TNF-alpha-receptor-like protein	all strains
39.	K3R	OPG213	TNF-alpha-receptor-like protein	all strains
40.	T1R	OPG214	Golgi antiapoptotic protein	all strains
41.	I2R	OPG004	ankyrin repeat-containing protein	all strains

^a^ As in CPXV strain Kostroma_2015 nomenclature. ^b^ As in Senkevich et al. nomenclature [47].

**Table 2:** List of MPXV genes under adaptive selection.

#	Gene Name ^a^	Gene Name ^b^	Function	Strains for Which Adaptive Selection Is Shown	Gene Name ^c^/Ref.
1.	C9L	OPG023	Host range, ankyrin repeat-containing protein	some isolates of the clade IIa MPXV	-/[61]
2.	C11L	OPG025	Host range, ankyrin repeat-containing protein	some isolates of the clade IIa MPXV	C9L/[61]
3.	C13L	OPG027	Interferon antagonist, host-range protein, likely involved in host immune evasion	CPXV when compared with clade II MPXV	C7L/[62]
4.	C16L	OPG031	IL-1 receptor antagonist	W-Nigeria when compared with clade I MPXV	C4L/[63]
5.	M6R	OPG044	Bcl-2-like protein which, through its interaction with the DEAD box RNA helicase DDX3X/DDX3, prevents TBK1/IKKepsilon-mediated IRF3 activation, contributes to virulence by binding to the host TRAF6 and IRAK2 and preventing host NF-kappa-B activation	clade I when compared with clade II MPXV	K7R/[64]
6.	G1L	OPG045	Caspase-9 inhibitor, protein with a BCL2-like fold which is essential for survival of infected cells.	some isolates of the clade IIa MPXV	F1L/[65]
7.	G3L	OPG047	Kelch-like protein, intracellular protein that affects the innate immune response	some isolates of the clade I MPXV	F3L/[66]
8.	A26L	OPG152	cowpox A-type inclusion protein	some isolates of the clade IIa MPXV	-/[67]
9.	A27L	OPG153	cowpox A-type inclusion protein	some isolates of the clade IIa MPXV	A26L/[67]
10.	A34R	OPG161	EEV membrane phosphoglycoprotein coordinates the incorporation of A36 into intracellular enveloped virion (IEV) membranes and, subsequently, the production of actin tails, therefore plays an essential role in efficient cell-to-cell spread of viral particles	some isolates of the clade IIa when compared with clade IIb MPXV	A33R/[68]
11.	A36R	OPG163	MHC class II antigen presentation inhibitor	some isolates of the clade IIa when compared with clade I MPXV	A35R/[69]
12.	A37R	OPG164	IEV transmembrane phosphoprotein	strain Zaire-96-I-16 when compared with clade I MPXV	A36R/[70]
13.	A45R	OPG172	Type-I membrane glycoprotein	some isolates of the clade IIa MPXV	A43R/[71]
14.	A46R	OPG173	Plays a role in the inhibition of host protein synthesis. Specifically, it inhibits the initiation of cap-dependent and cap-independent translation. In turn, it affects the outcome of infection by decreasing recruitment of inflammatory leukocytes and reducing the memory CD8+ T-cell response. Localizes in cytoplasmic structures that differ from virus factories.	CPXV when compared with clades I и IIa MPXV	-/[60]
15.	A58R	OPG185	Hemagglutinin protein is capable of binding two viral proteins, a serine protease inhibitor (K2) and the vaccinia virus complement control protein (VCP), and anchoring them to the surface of infected cells.	clade IIa when compared with clade IIb MPXV	A56R/[72]
16.	B5R	OPG191	Ankyrin repeat-containing protein	some isolates of the clade II when compared with clade I MPXV	B6R/[61]
17.	B11R	OPG198	Ser-Thr kinase protein, pseudokinase that plays a role in viral DNA replication repression by activating the antiviral protein BANF1 and inhibiting the activity of host VRK1, a cellular modulator of BANF1.	some isolates of the clade II when compared with clade I MPXV	B12R/[73]
18.	B17R	OPG204	Soluble interferon alpha/beta receptor protein	clade IIa when compared with clade IIb MPXV	B19R/[74]
19.	B20R	OPG208	Serine protease inhibitor (SPI-1), host-range protein, this viral protein may be involved in the regulation of the complement cascade	some isolates of the clade IIa when compared with clade IIb MPXV	C12L/[75]
20.	B21R	OPG209	Virulence protein, soluble TNF receptor II	clade IIa when compared with clade I MPXV	C13L/[63]
21.	B22R	OPG210	Putative membrane-associated glycoprotein	some isolates of the clades IIb и I MPXV	C14L/[63]
22.	I3R	OPG003	Ankyrin repeat-containing protein	some isolates of the clade IIa when compared with clade I MPXV	C19/[61]

^a^ As in CPXV strain Kostroma_2015 nomenclature. ^b^ As in Senkevich et al. nomenclature [47]. ^c^ As in VACV Copenhagen nomenclature.

## Data Availability

The original contributions presented in this study are included in the article/Supplementary Material. Further inquiries can be directed to the corresponding author(s).

## Supplementary Materials

The following supporting information can be downloaded at: https://www.mdpi.com/article/10.3390/v17121549/s1, Figure S1. Maximum likelihood tree based on multiple alignment of the central region of the MPXV genome. Table S1. Genes of the terminal left variable region of the CPXV genome strain Kostroma 2015. Table S2. Genes of the terminal right variable region of the CPXV genome strain Kostroma_2015.
