# Supplementary material for: Molecular Aspects of the Emergence of Monkeypox Virus Clades
Source: Viruses. 2025 Nov 26;17(12):1549. doi: 10.3390/v17121549 (PMC12737503; doi:10.3390/v17121549)
Supplement: Supplementary file 1 [file viruses-17-01549-s001.zip › viruses-3977278-supplementary.pdf]

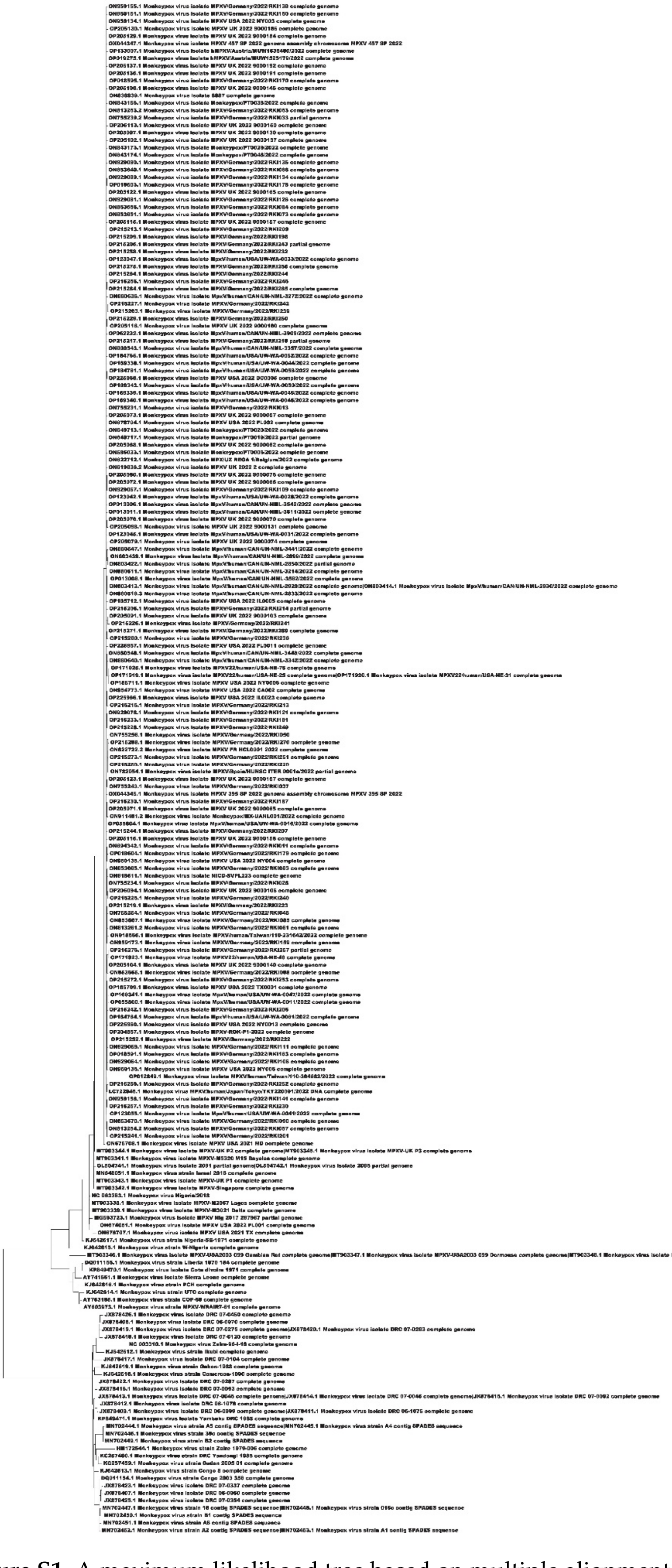

Figure S1. A maximum likelihood tree based on multiple alignment of the central region of the MPXV genome.

Table S1. Genes of the terminal left variable region of the CPXV genome strain Kostroma_2015.

#	Gene name [a]	Gene name [b]	Function
1.	D1L	OPG001	chemokine binding protein
2.	D2L	OPG002	Crm-B secreted TNF-alpha-receptor-like protein
3.	D3L	OPG003	ankyrin repeat-containing protein
4.	D4L	OPG004	ankyrin repeat-containing protein
5.	D5L	OPG005	Bcl-2-like protein
6.	D6L	OPG006	alpha-amanitin target protein
7.	D7L	OPG008	kelch-like protein
8.	D8L	OPG009	ankyrin repeat-containing protein
9.	D9L	-	C-type lectin-like protein
10.	D10L	-	C-type lectin domain-containing protein
11.	D11L	OPG011	kelch-like protein
12.	D12L	OPG012	TNF-alpha-receptor-like protein
13.	D13L	OPG013	TNF-alpha-receptor-like protein
14.	D14L	OPG014	ankyrin repeat-containing protein
15.	C1L	OPG015	ankyrin repeat-containing protein
16.	C2L	OPG016	MHC class I-like protein
17.	C3L	OPG017	ankyrin repeat-containing protein
18.	C4L	OPG018	host-range protein
19.	C5R	OPG019	epidermal growth factor-like protein (EGF-like protein)
20.	C6L	OPG020	IL-1 receptor antagonist
21.	C7R	OPG021	bifunctional zinc finger-like protein/E3 ubiquitin ligase
22.	C8L	OPG022	IL-18 binding protein
23.	C9L	OPG023	Host range ankyrin repeat-containing protein
24.	C10L	OPG024	retroviral pseudoprotease-like protein
25.	C11L	OPG025	host range ankyrin repeat-containing protein
26.	C12L	OPG026	Poxvirus_TNF-rcpt-II_C domain containing protein
27.	C13L	OPG027	host-range protein
28.	C14L	OPG029	Bcl-2-like protein
29.	C15L	OPG030	Kelch-like protein
30.	C16L	OPG031	IL-1 receptor antagonist
31.	C17L	OPG032	secreted complement-binding protein
32.	C18L	OPG033	kelch-like protein
33.	C19L	OPG034	hypothetical protein
34.	Q1L	OPG035	putative virulence factor
35.	Q2L	OPG036	putative alpha aminitin-sensitive protein
36.	P1L	OPG037	ankyin-like protein
37.	P2L	OPG038	NFkB inhibitor
38.	M1L	OPG039	ankyrin-like protein
39.	M2L	OPG040	serine protease inhibitor-like protein
40.	M3L	OPG041	interferon resistance protein
41.	M4L	OPG042	phospholipase-D-like protein
42.	M5L	OPG043	putative monoglyceride lipase
43.	M6R	OPG044	Toll/IL1-receptor, Bcl-2-like protein
44.	G1L	OPG045	caspase-9 inhibitor
45.	G2L	OPG046	dUTPase
46.	G3L	OPG047	Kelch-like protein

[a] As in CPXV strain Kostroma_2015 nomenclature

[b] As in Senkevich et. al. nomenclature [47]

Table S2. Genes of the terminal right variable region of the CPXV genome strain Kostroma_2015.

#	Gene name [a]	Gene name [b]	Function
1.	A25R	OPG151	DNA-dependent RNA polymerase subunit rpo132
2.	A26L	OPG152	cowpox A-type inclusion protein
3.	A27L	OPG153	cowpox A-type inclusion protein
4.	A28L	OPG154	IMV surface protein
5.	A29L	OPG155	IMV surface protein
6.	A30L	OPG156	DNA-dependent RNA polymerase rpo35
7.	A31L	OPG157	IMV protein
8.	A32R	OPG159	hypothetical protein
9.	A33L	OPG160	putative ATPase
10.	A34R	OPG161	EEV membrane phosphoglycoprotein
11.	A35R	OPG162	IEV and EEV membrane glycoprotein
12.	A36R	OPG163	MHC class II antigen presentation inhibitor
13.	A37R	OPG164	IEV transmembrane phosphoprotein
14.	A38R	OPG165	hypothetical protein
15.	A39R	OPG166	hypothetical protein
16.	A40L	OPG167	CD47-like membrane protein
17.	A41R	OPG168	semaphorin-like protein
18.	A42R	OPG169	C-type lectin-like type-II membrane protein
19.	A43L	OPG170	secreted glycoprotein
20.	A44R	OPG171	profilin-like protein
21.	A45R	OPG172	putative type-I membrane glycoprotein
22.	A46R	OPG173	inhibition of host protein synthesis.
23.	A47L	OPG174	hydroxysteroid dehydrogenase
24.	A48R	OPG175	Cu-Zn superoxide dismutase-like protein
25.	A49R	OPG176	Toll-IL1 receptor protein
26.	A50L	OPG177	immunoprevalent protein
27.	A51R	OPG178	thymidylate kinase
28.	A52R	OPG179	Bcl-2-like protein
29.	A53R	OPG180	ATP-dependent DNA ligase
30.	A54R	OPG181	hypothetical protein
31.	A55R	OPG182	Toll-IL receptor-like protein
32.	A56R	OPG183	secreted TNF-receptor-like protein
33.	A57R	OPG184	kelch-like protein
34.	A58R	OPG185	hemagglutinin
35.	A59R	OPG186	guanylate kinase
36.	B1R	OPG187	Ser-Thr kinase
37.	B2R	OPG188	Schlafen protein
38.	B3R	OPG189	ankyrin-like protein
39.	B4R	OPG190	EEV membrane glycoprotein
40.	B5R	OPG191	ankyrin-like protein
41.	B6R	OPG192	Virulence protein
42.	B7R	OPG193	soluble interferon-gamma receptor-like protein
43.	B8R	OPG195	ER-localized apoptosis regulator
44.	B9R	OPG196	Kelch-like protein
45.	B10R	OPG197	hypothetical protein
46.	B11R	OPG198	Ser-Thr kinase-like protein
47.	B12R	OPG199	inhibits Fas-mediated apoptosis, IL-1 convertase
48.	B13R	OPG200	Bcl-2-like protein

49.	B14R	OPG201	IL-beta-binding protein
50.	B15L	OPG202	IL-1-beta-inhibitor
51.	B16R	OPG203	ankyrin-like protein
52.	B17R	OPG204	soluble interferon alpha/beta receptor
53.	B18R	OPG205	ankyrin repeat-containing protein
54.	B19R	OPG206	Kelch-like protein
55.	B20R	OPG208	serine protease inhibitor-like protein (Serpin)
56.	B21R	OPG209	Virulence protein, soluble TNF receptor II
57.	B22R	OPG210	putative membrane-associated glycoprotein
58.	K1R	OPG211	ankyrin repeat containing protein
59.	K2R	OPG212	TNF-alpha-receptor-like protein
60.	K3R	OPG213	TNF-alpha-receptor-like protein
61.	T1R	OPG214	Golgi antiapoptotic protein
62.	I1R	OPG005	Bcl-2-like protein
63.	I2R	OPG004	ankyrin repeat-containing protein
64.	I3R	OPG003	ankyrin repeat-containing protein
65.	I4R	OPG002	Crm-B secreted TNF alpha receptor-like protein
66.	I5R	OPG001	chemokine binding protein

[a]As in CPXV strain Kostroma_2015 nomenclature

[b]As in Senkevich et. al. nomenclature [47]
